# In-the-Bag Intraocular Lens Placement via Secondary Capsulorhexis with Radiofrequency Diathermy in Pediatric Aphakic Eyes

**DOI:** 10.1371/journal.pone.0062381

**Published:** 2013-04-24

**Authors:** Lixia Luo, Haotian Lin, Weirong Chen, Chunxiao Wang, Xinyu Zhang, Xiangchen Tang, Jianping Liu, Nathan Congdon, Jingjing Chen, Zhuoling Lin, Yizhi Liu

**Affiliations:** State Key Laboratory of Ophthalmology, Zhongshan Ophthalmic Center, Sun Yat-sen University, Guangzhou, China; University of Durham, United Kingdom

## Abstract

Pediatric ophthalmologists increasingly recognize that the ideal site for intraocular lens (IOL) implantation is in the bag for aphakic eyes, but it is always very difficult via conventional technique. We conducted a prospective case series study to investigate the success rate and clinical outcomes of capsular bag reestablishment and in-the-bag IOL implantation via secondary capsulorhexis with radiofrequency diathermy (RFD) in pediatric aphakic eyes, in which twenty-two consecutive aphakic pediatric patients (43 aphakic eyes) enrolled in the Childhood Cataract Program of the Chinese Ministry of Health were included. The included children underwent either our novel technique for secondary IOL implantation (with RFD) or the conventional technique (with a bent needle or forceps), depending on the type of preoperative proliferative capsular bag present. In total, secondary capsulorhexis with RFD was successfully applied in 32 eyes (32/43, 74.4%, age 5.6±2.3 years), of which capsular bag reestablishment and in-the-bag IOL implantation were both achieved in 30 eyes (30/43, 70.0%), but in the remaining 2 eyes (2/32, 6.2%) the IOLs were implanted in the sulcus with a capsular bag that was too small. Secondary capsulorhexis with conventional technique was applied in the other 11 eyes (11/43, 25.6%, age 6.9±2.3 years), of which capsular bag reestablishment and in-the-bag IOL implantation were both achieved only in 3 eyes(3/43, 7.0%), and the IOLs were implanted in the sulcus in the remaining 8 eyes. A doughnut-like proliferative capsular bag with an extensive Soemmering ring (32/43, 74.4%) was the main success factor for secondary capsulorhexis with RFD, and a sufficient capsular bag size (33/43, 76.7%) was an additional factor in successful in-the-bag IOL implantation. In conclusion, RFD secondary capsulorhexis technique has 70% success rate in the capsular bag reestablishment and in-the-bag IOL implantation in pediatric aphakic eyes, particularly effective in cases with a doughnut-like, extensively proliferative Soemmering ring.

## Introduction

Pediatric cataract patients are commonly left aphakic after lens removal in early childhood, particularly when surgery is performed in the first year of life.[1]–[3] Secondary intraocular lens (IOL) implantation is considered when an aphakic child is unable to tolerate contact lenses or glasses or when more functional vision without additional correction is desired. [4] Although the ciliary sulcus was the common site of implantation for many years, pediatric ophthalmologists increasingly recognize that the ideal site for IOL implantation is in the bag. In-the-bag implantation sequesters the IOL from the highly reactive uveal tissue and maintains better IOL centration. [5], [6].

In many parts of China, pediatric cataract patients do not receive standardized treatment and regular follow-up, possibly because health resources are limited and patient compliance is poor. [7] Clinically, we have encountered serious challenges attempting secondary IOL implantation via conventional techniques in pediatric aphakic eyes, which are commonly characterized by severely fibrotic, fused anterior-posterior-capsulotomy edges and a doughnut-like Soemmering ring after many years without follow-up. In some cases, this ring of intracapsular lens material can become quite bulky, leaving less space in the ciliary sulcus for IOL placement and causing inevitable iris chafing and secondary glaucoma. [8], [9] In the past, we most often used a bent needle or forceps for secondary capsulorhexis and reopened the fibrotic, closed capsular bag in an attempt to achieve in-the-bag IOL placement. [10] However, it was not always easy to control the direction of the tear or to complete a continuous curvilinear capsulorhexis. Under these circumstances, when the IOL was placed into the bag, the anterior capsule was likely to tear.

The surgical treatment of pediatric cataracts is constantly changing as a result of advances in the armamentarium of surgeons and microsurgical techniques. Capsular surgery using radiofrequency diathermy (RFD) was originally described by Kloti in 1984 [11] and further examined by Gassmann and colleagues in 1988. [12] Although the RFD technique has been commercially available and in current clinical use for a number of years, we are unaware of any studies that have used RFD for secondary capsulorhexis in pediatric aphakic eyes. [2], [3].

In this paper, we present a valuable new procedural addition to RFD that allows the removal of a fibrotic capsule and the reopening of the capsular bag for in-the-bag IOL implantation using RFD. Then, we investigated the success rate and success factors of our novel surgical technique associated with capsular bag reestablishment and in-the-bag IOL implantation in pediatric aphakic eyes with proliferative capsular bags and report the preliminary clinical outcomes of the procedure.

## Materials and Methods

### Patients

Pediatric patients were selected from the Childhood Cataract Program of the Chinese Ministry of Health (CCPMOH), which initiated a series of ongoing studies on the influence of early interventions on the long-term outcomes of pediatric cataract treatment. [7] The study was approved by the institutional review board of Zhongshan Ophthalmic Center (ZOC), Guangzhou, China. Informed written consent was obtained from at least one parent of each participating child, and the tenets of the Declaration of Helsinki were followed throughout this study.

Pediatric patients were included in this study if they had aphakic eyes after previous cataract surgery and were scheduled to undergo secondary IOL implantation. There was some variation in the surgical technique used for the previous cataract surgery, which was a function of the patient’s age, the year of surgery and the degree of posterior capsule opacity. In the previous surgeries, a limbal or scleral tunnel incision was employed, and an anterior capsulotomy was made in a continuous curvilinear manner. The nucleus and cortex were removed using an irrigation/aspiration (I/A) device or an automated vitrectomy instrument, the latter of which was used to create a central posterior capsulotomy and perform a limited anterior vitrectomy in select cases. Peripheral iridectomy was rarely performed.

However, all of the aphakic eyes included in the study were required to have adequate residual capsular support (the diameter of previous posterior capsulotomy≤5 mm) and a capsular bag that was completely closed with anterior-posterior synechia and filled with proliferative cortical material and/or an opaque membrane, which were evaluated with a BX 900® Photo Slit Lamp (Haag-Streit AG, Switzerland) and a Pentacam Scheimpflug Imaging System (Oculus, Germany) before surgery. [13] Furthermore, all of the included children were required to attend all of the follow-up appointments specified in our protocol. Children who were noncompliant with our follow-up protocol were excluded before data collection began.

### Surgical Technique (Video S1)

The patients underwent general anesthesia for the surgeries, which were all performed by the same surgeon (Y.L.). The included eyes underwent secondary IOL implantation using either our novel technique (secondary capsulorhexis with RFD) or the conventional technique (secondary capsulorhexis with bent needle or forceps). Our novel surgical technique would be first tried to be applied in the included eyes. However, the conventional technique would be chosen if there was a high risk of unexpected thermal burns of posterior capsule by the RF tip during anterior capsulorhexis. In-the-bag IOL implantation was attempted in all included eyes after the cortical material was removed if the size of peripheral capsular bag was large enough (diameter >8 mm) without capsular tear during anterior capsulorhexis.

**Figure 1:**
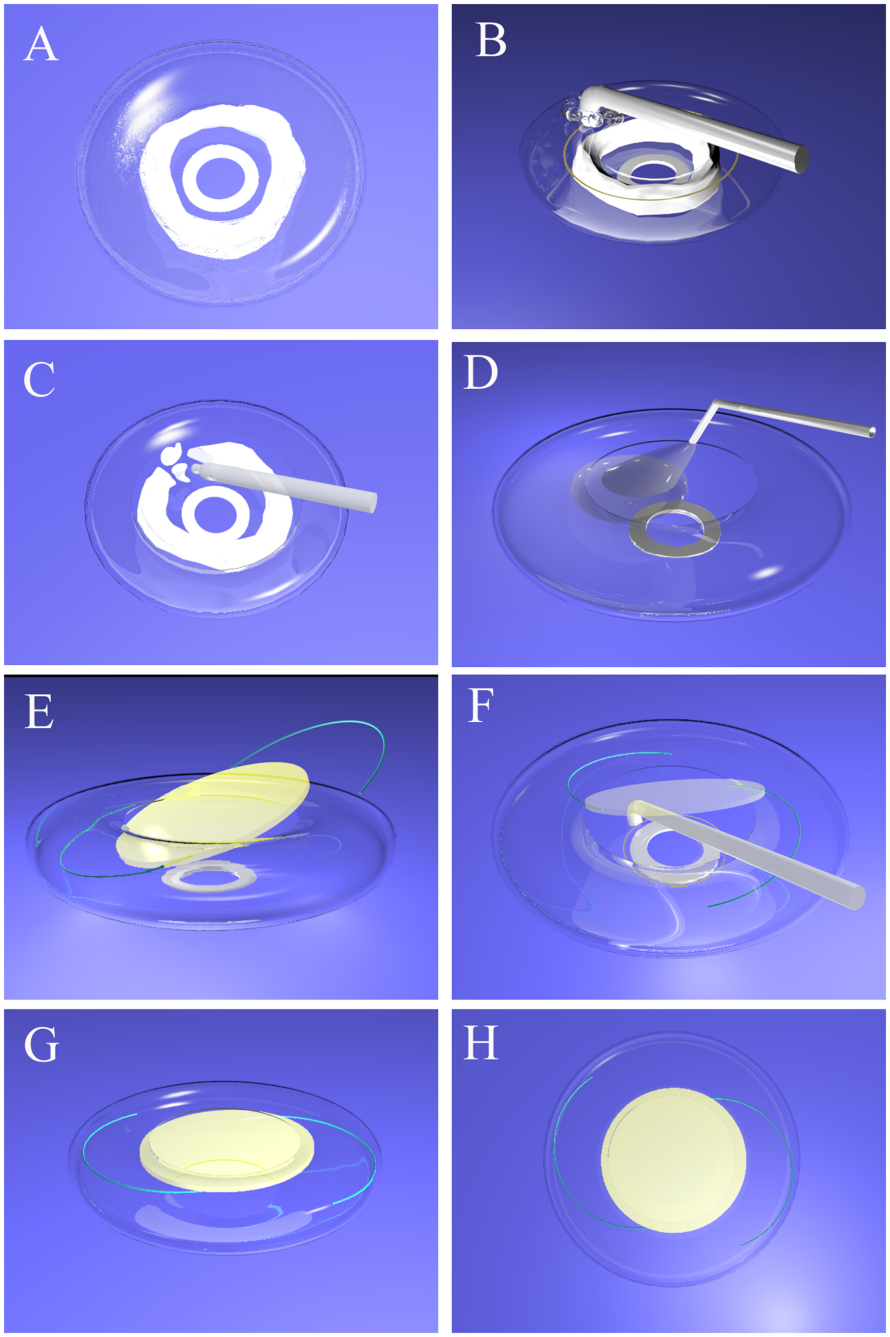
Schematic diagrams of the surgical techniques of capsular bag reestablishment and in-the-bag IOL implantation via secondary capsulorhexis with RFD. A, We used sodium hyaluronate to maintain the anterior chamber of an aphakic eye with a doughnut-like, extensive Soemmering ring. B, The RF tip was inserted into the anterior chamber to cut the anterior fibrotic capsule with coagulation energy, and the appearance of a visible bubble line indicates the cutting site. C, The cortical material was removed via I/A or a phaco tip using a divide-and-conquer approach. D, The capsular bag was reestablished with sodium hyaluronate. E, An IOL was implanted into the peripheral capsular bag. F, The RF tip was inserted into the anterior chamber again through the tilted IOL to cut the posterior fibrotic capsule. G, The IOL was dialed centrally after secondary capsulorhexis of the posterior capsule. H, The IOL was well-centered and implanted in the bag after the sodium hyaluronate was cleared. Notes: RFD = radiofrequency diathermy, IOL = intraocular lens, I/A = irrigation/aspiration.

Briefly, our novel surgical technique was as follows (Figure 1). After a conjunctival peritomy was made, a 3.2-mm superior scleral tunnel incision was created. We then used sodium hyaluronate to maintain the anterior chamber, and the RF tip (Oertli Instrumente, AG, Switzerland) was inserted into the anterior chamber through the incision. The applied coagulation energy (setting: high energy mode, 500 kHz) easily cut the anterior fibrotic capsule without resistance or complications, but the energy could also burn the posterior capsule if there was not enough cortical material between anterior and posterior capsule. The appearance of a visible bubble line indicated the cutting site. After the cortical material was removed with the I/A device, we implanted an IOL in the peripheral capsular bag. Our IOL of choice for secondary in-the-bag IOL placement is a 3-piece (Sensar AR40e, AMO, Inc. CA, USA) or 1-piece (Acrysof SA60AT, Alcon Laboratories, Inc. Texas, USA) acrylic IOL with a 6.5-mm optic at random, but only 1-piece (Acrysof SA60AT, Alcon Laboratories, Inc. Texas, USA) acrylic IOL was used for sulcus IOL placement, each of which was inserted with an appropriate specialized injector. The subsequent steps included (secondary) capsulorhexis of the posterior capsule with RFD, anterior vitrectomy, irrigation and aspiration. The tunnel incision was sutured with 10-0 nylon sutures. All of the patients received subconjunctival dexamethasone (2 mg) before the surgery was completed.

The conventional technique followed the method described above, with the important exception that a bent needle or forceps was used for the secondary capsulorhexis, which is the standard step recommended in the literature. [10].

### Postoperative Medicine Administration Regimen

The standard postoperative regimen consisted of tobradex eye drops (tobramycin 0.3%, dexamethasone 0.1%, Alcon) 4 times per day and tobradex eye ointment (tobramycin 0.3%, dexamethasone 0.1%, Alcon) once per night for 1 month. Subsequently, anti-inflammatory steroid drugs were replaced with a steroid-free drug for another month (pranoprofen eye drops, 4 times per day, Senju Pharmaceutical Co., Ltd. JP). If the intraocular pressure (IOP) was ≥21 mmHg, additional antiglaucoma drugs were administered according to the regimen followed in our previous study. [14].

### Follow-up Protocol and Assessment Methods

The follow-up appointments were scheduled according to our previous protocol. [7] Specifically, in this study, follow-up appointments consisted of orthoptic assessment, IOP measurement using a Tono-Pen tonometer (Reichert Inc., Seefeld, Germany), and fundoscopy, which included an optic disc assessment. Concurrently, the degree of anterior segment inflammation and the stability of the IOL were examined with a BX 900® Photo Slit Lamp (Haag-Streit AG, Switzerland) and a Pentacam Scheimpflug Imaging System (Oculus, Germany) at every follow-up visit and documented. The slit lamp photographic images were subjectively evaluated for inflammation and IOL stability, and the software included on the Pentacam device automatically and quantitatively graded the Pentacam images with respect to corneal edema and the stability of the IOL. [15], [16] Because young children have poor compliance with examination, we have developed techniques and equipment specific for pediatric ophthalmic examinations. [14] We performed examinations after administering a sedative drug (i.e., chloral hydrate as a sleep aid) or under general anesthesia (if chloral hydrate was contraindicated or unacceptable) for children who were too uncooperative to allow for regular examinations in the clinic.

### Main Outcome Measures

The success rate of secondary capsulorhexis with RFD/in-the-bag IOL placement was defined as the proportion of successful secondary capsulorhexis with RFD/in-the-bag IOL placement among the included cases. Intraoperative complications can include capsular tear, iris or corneal thermal burns by the RF tip, and the presence of residual proliferative material in the vitreous cavity. Postoperative complications can include ocular hypertension (IOP≥21 mmHg), corneal edema, delayed healing of the corneal incision, increased postoperative inflammation, iris synechia, pigmentation dispersion, macular edema, retinal detachment, recurrence of severe tissue reactivity and shrinkage of the capsule. The axial stability of in-the-bag IOL was evaluated with the Pentacam Scheimpflug Imaging System. IOL decentration was recorded using slit lamp photographic images if the edge of the IOL optic could be visualized through the undilated pupil or if the IOL/capsular bag complex appeared decentered. However, the in-the-bag IOL placement and rotation stability were evaluated by comparing slit lamp photographic images after pupil dilation at different follow-up appointments. [16].

### Statistical Analysis

Continuous variables were expressed as means ± standard deviations (SD), whereas categorical data were expressed as numbers (n) and percentages (%). Because of the small sample size, Mann-Whitney tests were used to identify differences between our novel technique and the conventional technique. Categorical variables were compared using Fisher’s exact test. To look at the factors influencing on surgical success of capsular bag reestablishment and in-the-bag IOL implantation, the type of Soemmering ring, capsular bag size, and age were statistical analyzed between the cases via our novel technique and conventional technique. All statistical tests were two-sided, and the 0.05 level was considered statistically significant. Data were analyzed using SPSS 17.0 (SPSS Inc., Chicago, IL) for Windows.

## Results

**Figure 2:**
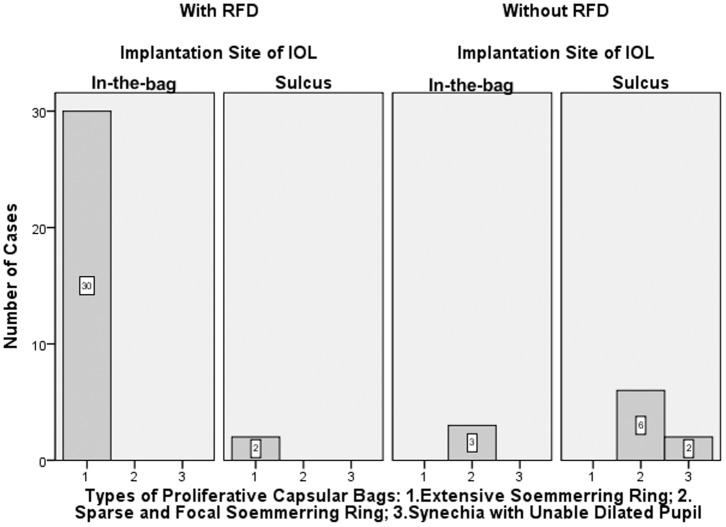
The relationships between different types of proliferative capsular bags, secondary capsulorhexis with/without RFD and the IOL implantation site in two groups. The number of cases is shown in different columns. Proliferative capsular bag Type 1 was included in Group A, and proliferative capsular bag Types 2 and 3 were included in Group B. Notes: RFD = radiofrequency diathermy, IOL = intraocular lenses.

A total of 43 aphakic eyes of 22 children (age: 6.0±2.3 years), including 17 males (17/22, 77.3%) and 21 children (21/22, 95.5%) with bilateral cataracts, were consecutively recruited from CCPMOH from December 2010 to March 2012. However, 2 children (4 eyes via our novel technique, 4/43, 9.3%) were excluded from analysis of postoperative complications for noncompliance with our follow-up protocol. Altogether (Figure 2), secondary capsulorhexis with RFD was successfully applied in 32 aphakic eyes (32/43, 74.4%, age 5.6±2.3 years), of which capsular bag reestablishment and in-the-bag IOL implantation were also achieved in 30 eyes (30/32, 93.8%), and the IOLs were implanted in the sulcus with a capsular bag that was too small in the remaining 2 eyes (2/32, 6.2%). Secondary capsulorhexis with conventional technique was applied in the other 11 aphakic eyes (11/43, 25.6%, age 6.9±2.3 years), of which capsular bag reestablishment and in-the-bag IOL implantation were both achieved only in 3 eyes (3/11, 27.3%, p<0.05 compared with cases via our novel technique), and the IOLs were implanted in the sulcus in the remaining 8 eyes (1 eye with unexpected tear in posterior capsule, 3 eyes with small capsular bag size and failure of capsular bag reestablishment in the other 4 eyes because of close adhesion of residual anterior-posterior-capsule). It is necessary to mention that one of the 11 aphakic eyes via conventional technique was initially applied with our novel RFD technique, but the RFD technique was replaced by the conventional technique because of timely detection of the unexpected thermal burns of posterior capsule by the RF tip during anterior capsulorhexis, resulting in sulcus IOL implantation.

**Figure 3:**
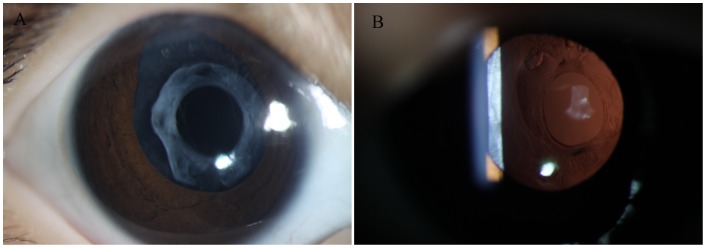
Photograph of an aphakic eye with a proliferative capsular bag. A, The capsular bag was completely closed with anterior-posterior synechia, was filled with proliferative cortical material and had an extensive Soemmering ring. B, The capsular bag was completely closed with anterior-posterior synechia, but it had little cortical material and a sparse and focal Soemmering ring.

There was a significant correlation between the types of preoperative proliferative capsular bag present and the successful application of secondary capsulorhexis with RFD. If the proliferative capsular bag had an extensive/continuous Soemmering ring (Figure 3A), this type of capsular bag could be successfully applied with our novel surgical technique, although in-the-bag IOL placement in this type of closed, fibrotic capsular bag is difficult to accomplish with the traditional armamentarium and methods. If the aphakic eyes were with a proliferative capsular bag, a sparse/focal Soemmering ring or synechia and an undilatable pupil (Figure 3B), our novel surgical technique was unsuitable because of easily unexpected thermal burn of posterior capsule and should be instead with the conventional technique. The reestablishment of this type of proliferative capsular bag is always difficult. In all, we found that a doughnut-like proliferative capsular bag with an extensive Soemmering ring (32/43, 74.4%) was the main success factor for secondary capsulorhexis with RFD. A sufficient capsular bag size (33/43, 76.7%) was an additional factor in successful in-the-bag IOL implantation. However, age was found not to be a factor influencing on surgical success of capsular bag reestablishment and in-the-bag IOL implantation.

**Figure 4:**
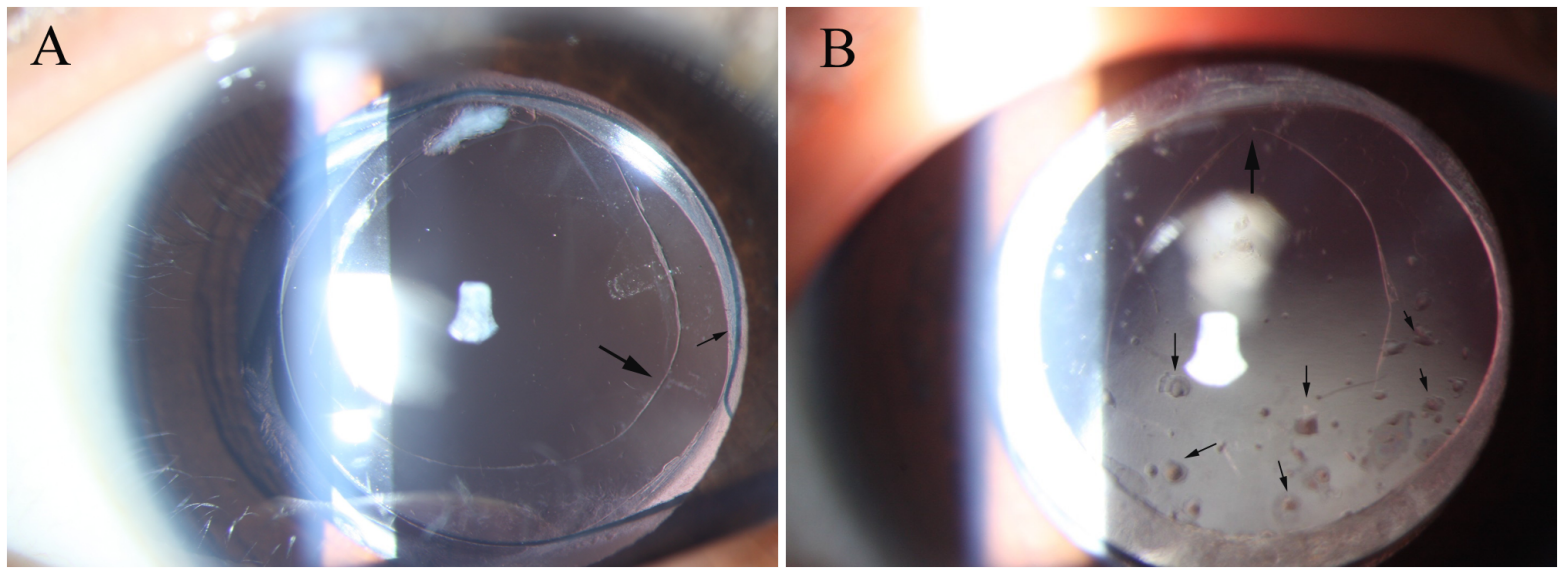
Postoperative photographs of secondary IOL implantation 1 week after surgery. A, The in-the-bag IOL was centered in the peripheral capsular bag with clear anterior (small arrow) and posterior (large arrow) secondary capsulorhexis relative to the smooth edge in one of the eyes underwent our novel surgical technique. B, In-the-bag IOL was accompanied by epithelium proliferation (small arrow) scattered in the visual axis and a relatively rough posterior edge (large arrow) after secondary capsulorhexis in one of the eyes underwent the traditional surgical technique. Notes: IOL = intraocular lenses.

All Soemmering ring material and proliferative membranes in the visual axis were intraoperatively removed without capsular tear in all included eyes, except the stated case of unexpected thermal burns of posterior capsule during anterior capsulorhexis initially by the RF tip. No iris or corneal thermal burns from the RF tip occurred and no residual proliferative material was present in the vitreous cavity during the operation. One week after surgery, the in-the-bag IOL was centered in the peripheral capsular bag with a clear and relatively smooth-edged secondary capsulorhexis in eyes via our novel technique. In eyes via conventional technique, however, the IOL implantation was accompanied by epithelium proliferation scattered in the visual axis and a relatively rough posterior edge after secondary capsulorhexis (Figure 4).

**Figure 5:**
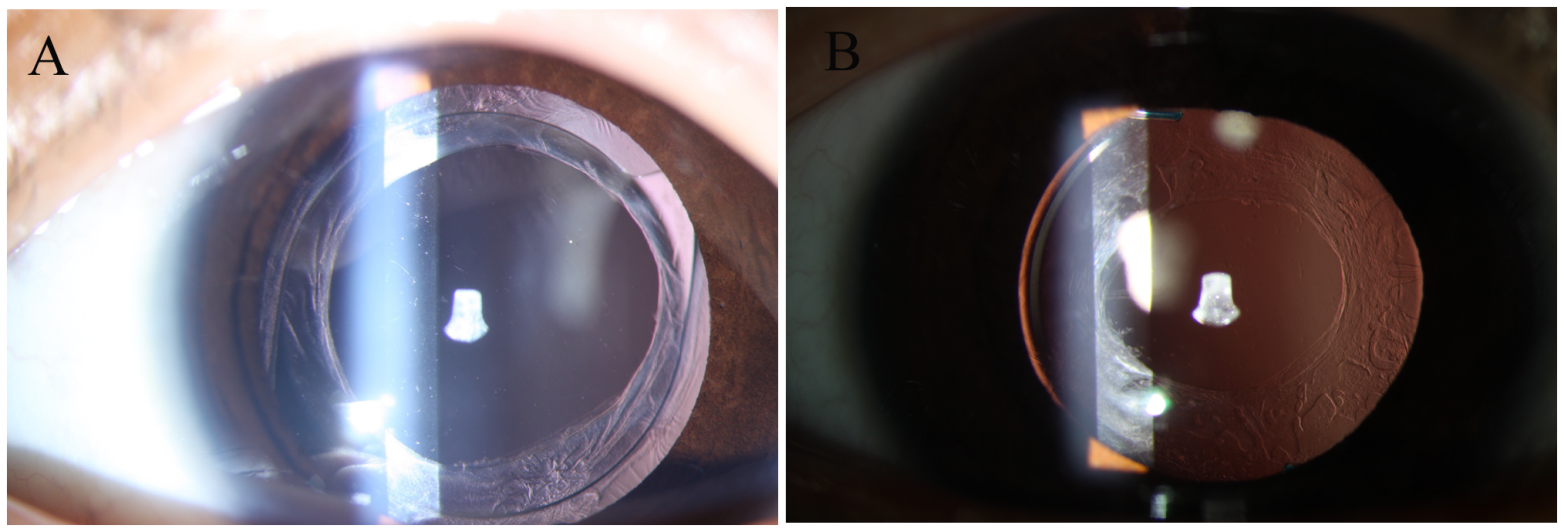
Postoperative photograph of secondary IOL implantation 3 months after surgery. A, The in-the-bag IOL was well-centered in the peripheral capsular bag without any epithelium proliferation in the visual axis in one of the eyes underwent our novel surgical technique. B, Apparent epithelium proliferation and shrinkage of the residual capsular bag was identified in one of the eyes underwent the traditional surgical technique. Notes: IOL = intraocular lenses.

Throughout the postoperative follow-up period (13.3±4.6 months), no corneal edema, delayed corneal incision healing, or inflammatory reaction was found to have been triggered by residual material or the surgical intervention. Furthermore, no iris synechia, pigmentation dispersion, macular edema, retinal detachment, recurrence of severe tissue reactivity or capsule shrinkage were identified. However, ocular hypertension (IOP≥21 mmHg) occurred in 5 eyes (5/28, 17.9%) via our novel technique and 2 eyes (2/11, 18.2%) via conventional technique. Lens epithelium proliferation in the visual axis was identified in 4 eyes via conventional technique but no eyes via our novel technique (Figure 5). No IOL displacement (decentration, rotation or tilt) was discovered in any of the included eyes upon analysis with the Pentacam Scheimpflug Imaging System or upon comparing the slit lamp photographic images after pupil dilation at different follow-up appointments.

## Discussion

In this paper, we presented a new procedural addition to RFD that will be valuable to any clinician responsible for the care of pediatric cataract patients with challenging cases. The procedure involves the removal of a fibrotic capsule and reopening of the capsular bag with RFD for in-the-bag IOL implantation in pediatric aphakic eyes. We recruited 22 consecutive aphakic pediatric patients (43 aphakic eyes) from CCPMOH and conducted this prospective case series study to investigate the success rate and clinical outcomes of novel technique. We found that a high success rate (70%) of capsular bag reestablishment and in-the-bag IOL implantation could be easily achieved with RFD secondary capsulorhexis in pediatric aphakic eyes, particularly those with a doughnut-like, extensively proliferative Soemmering ring. Furthermore, we found significantly decreased lens epithelium proliferation after secondary IOL implantation with RFD secondary capsulorhexis than the conventional technique in pediatric aphakic eyes.

It is well known that the technical ease and success of secondary IOL implantation depends on the capsular support that remains after primary cataract surgery. [13] However, secondary IOL insertions in aphakic pediatric patients present considerable challenges, especially in the presence of severe tissue reactivity, capsular shrinkage and Soemmering ring formation. [17] These complications increase the risk of capsular tear and may lead to the inadvertent sulcus placement of the IOL, which can lead to such additional complications as IOL displacement, pigmentation dispersion and iris damage. [8].

An increasing number of pediatric ophthalmologists have recognized that the ideal site for IOL implantation is in the bag. This placement sequesters the IOL from the highly reactive uveal tissue and maintains better IOL centration. However, in-the-bag secondary IOL implantation presents a challenge in pediatric aphakic eyes with a closed fibrotic capsule bag. [2] In the past, when we performed secondary capsulorhexis to reestablish the fibrotic capsular bag in aphakic patients, we typically used a bent needle and/or forceps. Using this technique, it was always difficult to control the tear direction, and it was difficult to complete a continuous curvilinear capsulorhexis. Early in 1999, Wilson et al. [18] first described a technique for achieving in-the-bag secondary implantation in children. The authors used a vitrector probe to circumferentially cut close to the point of fusion of the anterior and posterior capsules, and they then used a vitrector to remove the Soemmering ring material. In 2008, Gimbel and Venkataraman [19] described an adult patient in whom they performed secondary IOL placement. An anterior capsulotomy was performed using a cystotome, and the Soemmering ring was aspirated using a manual Simcoe cannula, after which the IOL was inserted in the bag. Recently, Dilraj et al. [20] reported a modified technique that was built on previous techniques for Soemmering ring removal in aphakic eyes, followed by the in-the-bag placement of a secondary IOL. Unique to their technique is the use of 20-gauge MST scissors to cut the anterior capsule to complete the anterior capsulotomy, as well as a 2-handed technique to manually fragment and debulk the Soemmering ring, followed by the viscoexpression of large fragments in Dilraj’s study [20]. However, all of the described techniques in the previous studies are somewhat complex, and their effectiveness and safety profiles were not reported because of the lack of a well-conducted prospective case series study. Furthermore, we are not aware of any studies that have used the RFD technique for secondary capsulorhexis to achieve secondary IOL in-the-bag implantation in pediatric aphakic eyes, despite the fact that the RFD technique has been commercially available and in current clinical use for many years.

Capsulorhexis using RFD was originally reported by Kloti [11] and further researched by Gassmann [12]. With RFD, the capsule is cut and coagulated using a platinum-alloy-tipped probe heated to approximately 160°C by a modulated, high-frequency current of 500 kHz. Although diathermy capsulorhexis techniques have achieved good results in particular clinical situations, [21]–[23] these techniques are not popular in cataract cases because they offer less elasticity and resistance. [24] The negative reports of decreased capsular elasticity and the lack of an obvious advantage in adult primary capsulorhexis using RFD compared with using traditional forceps may explain why research on the RFD technique has stalled. It is well known that the pediatric capsule is highly elastic, making it difficult to perform capsulorhexis with a bent needle or forceps. Moreover, in secondary IOL implantation cases, it is difficult to distinguish the anterior opening even with capsule staining because of the white proliferative cortical material between the anterior and posterior capsular leafs. However, RFD is easy to perform in this situation, regardless of the proliferative anterior capsular visibility and resistance. In our study, we found that the RF tip could be especially easily controlled to create a highly predictable secondary capsulorhexis in severely proliferative aphakic eyes with a doughnut-like residual capsule. Interestingly, the challenging condition of a doughnut-like proliferative capsular bag with extensive Soemmering ring formation for secondary IOL implantation, either in the bag or in the sulcus, happened to be a critical success factor for the application of our new RFD procedure (reduced risk of unexpected thermal burns of posterior capsule by the RF tip during anterior capsulorhexis); however, studies with a larger sample size are still warranted to further explore the application of our novel technique.

Most patients who present for secondary IOL insertion have a central posterior capsulotomy. In these eyes, inadvertent partial or complete loss of the Soemmering ring into the vitreous cavity is possible when the Soemmering ring is removed using the techniques described in previous studies. Dilraj et al. [20] believed that performing all of the maneuvers for Soemmering ring removal in an ophthalmic viscoelastic-filled eye minimized the risk of Soemmering ring loss. Although Dilraj’s technique had the advantage of avoiding the active infusion of the anterior chamber with balanced salt solution if necessary as a vitrector, the use of a Simcoe cannula or phacoemulsification probe to remove Soemmering ring material presented no advantages over the techniques described by Wilson et al. [18] and Gimbel and Venkataraman [19], and there were no reports of the intraoperative complications resulting from the technique in Dilraj’s report. In the present study, we performed secondary capsulorhexis of the anterior proliferative capsular bag (Figure 1A) and then immediately cleared all doughnut-like, extensively proliferative material and the Soemmering ring (Figure 1C), which was still separated from the vitreous cavity before we performed the secondary capsulorhexis of the posterior proliferative capsular (Figure 1F). In all of the 32 eyes in which RFD was successfully applied, no loss of residual proliferative material into the vitreous cavity occurred during the operation, which showed that our procedure had great effectiveness and excellent safety. Furthermore, no iris or corneal thermal burns due to the RF tip occurred during surgery, which contributed to the excellent safety of our technique.

It is a widely recognized fact that active lens epithelial cells remain after cataract surgery in almost all pediatric cataract patients, despite improvements in surgical techniques, such as hydrodissection and vacuuming of the capsular bag. [2] In our study, we noted that congenital or infantile cataracts in the first year of life have a much more exuberant cortex deposition within the capsular bag than those in children operated on at a later age, which was similar to the findings of Wilson et al. [18] This lens material always forms the extensive Soemmering ring (constituting 74.4% of the eyes in our study). If the ring is sparse, in focal areas and enclosed between the anterior and posterior capsules (constituting 25.6% of the eyes in our study), it may be reasonable to leave it unopened at the time of secondary IOL implantation. However, if the Soemmering ring is extensive, sulcus placement of the IOL risks future IOL tilt and uveitis-glaucoma-hyphema syndrome with subsequent enlargement of the Soemmering ring. [25] Although in-the-bag IOL implantation was not achieved in 2 eyes via our novel technique in this study (while sulcus IOL implantation was achieved) because of the small size of the capsular bag, the extensive proliferative material and Soemmering ring were completely cleared after RFD secondary capsulorhexis, with no signs of IOL tilt or uveitis-glaucoma-hyphema syndrome during our postoperative follow-up. Additionally, the other eyes in our study demonstrated good clinical outcomes without the above mentioned posterior complications.

Most pediatric ophthalmologists suggest that if the anterior capsule overlying the Soemmering ring material is opened, it is best not to leave any Soemmering ring material, as residual material may trigger an inflammatory reaction. [19] In our study, all Soemmering ring material was completely removed intraoperatively in all included eyes, which was indirectly proven by the fact that no secondary inflammation reaction was identified during our follow-up. Although ocular hypertension (IOP≥21 mmHg) occurred in 5 eyes (5/28, 17.9%) via our novel technique and 2 eyes (2/11, 18.2%) via conventional technique, all of the cases of ocular hypertension were self-limiting and presented with steroid-induced characteristics, which are discussed in another study. [14].

Unique to our technique is the use of an RF tip to complete the secondary capsulorhexis of the anterior and posterior capsules separately, which not only exposes the proliferative Soemmering ring material for clearing but also prevents the loss of residual fragments into the vitreous cavity. At the same time, both the anterior and posterior capsular fibrosis at the rhexis edge can be circumferentially cut, and continuous secondary capsulorhexis can be precisely achieved without the risk of a radial capsule tear, which can help maintain IOL stability in the bag. Moreover, swollen capsular edges have been reported after diathermy capsulotomy, such that the lens epithelium was less proliferative and less capsule shrinkage occurred [26]. Interestingly, we found in the current study that lens epithelium proliferation in the visual axis was identified in 4 eyes (4/11, 36.4%) via conventional technique, but in no eyes via our novel technique. We believe that the diathermy used during capsulorhexis, which is unique to our technique, could help to decrease the number of residual active lens epithelial cells and thus prevent long-term epithelium proliferation. A randomized controlled study with a larger sample size is needed to investigate whether there is a significant difference between the occurrence rates of post-operative residual lens epithelium proliferation and the incidence of secondary IOL tilt and decentration due to extensive proliferation.

The results and interpretation of the present study must be understood within the context of the study’s strengths and limitations. The study’s strengths include its strict, consistent follow-up protocol; the use of reasonably accurate measurements within a national research program (CCPMOH); the first presentation of a valuable new addition to RFD; the first classification of an extensive/continuous Soemmering ring, sparse/focal Soemmering ring, and synechia with and undilatable pupil as three types of proliferative capsular bags; the first clinical outcomes report of this new technique compared with the conventional bent needle or forceps technique; and the first report of the success rate and the first discussion of the success factors for the RFD technique. The study weaknesses must also be acknowledged. Although this study was included in a national program, all of the subjects underwent surgery and were followed at a single center without a randomized and controlled design. Therefore, the results should be confirmed and the indications for the application of RFD secondary capsulorhexis in pediatric aphakic eyes should be further defined in future studies that include large sample sizes with a randomized and controlled design and long-term follow-up.

Despite these limitations, the current study remains one of the first to report that a high success rate of capsular bag reestablishment and in-the-bag IOL implantation could be easily achieved with RFD secondary capsulorhexis in pediatric aphakic eyes with a doughnut-like, extensively proliferative Soemmering ring. The simplicity, effectiveness, and excellent safety profiles of this procedure make it a valuable addition to the armamentarium of any clinician responsible for these challenging patient cases.

## Supporting Information

Video S1 **Surgical techniques of in-the-bag IOL implantation in pediatric aphakic eyes.**
(WMV)
